# Hexaaqua­cobalt(II) 5,5′-(propane-1,3-diyldithio)bis­(1*H*-tetra­zole-1-acetate)

**DOI:** 10.1107/S1600536809028463

**Published:** 2009-07-29

**Authors:** Wan-Ling Liang, Qing Yu, Xiu-Qing Zhang, Jiang-Ke Qin, Hong Liang

**Affiliations:** a College of Chemistry and Chemical Engineering, Guangxi Normal University, Guilin, Guangxi 541004, People’s Republic of China

## Abstract

The asymmetric unit of the title complex, [Co(H_2_O)_6_](C_9_H_10_N_8_O_4_S_2_), contains one-half of a [Co(H_2_O)_6_]^2+^ cation and one-half of a 5,5′-(propane-1,3-diyldithio)bis­(1*H-*tetra­zole-1-acetate) (battp^2−^) anion. The Co^II^ center is coordinated by six H_2_O mol­ecules in a distorted octa­hedral coordination environment. In the crystal structure, intra- and inter­molecular O—H⋯O and O—H⋯N hydrogen bonds link the cations and anions into a three-dimensional network. π–π contacts between the tetra­zole rings [centroid–centroid distance = 3.346 (1) Å] may further stabilize the structure.

## Related literature

For related structures, see: Du *et al.* (2004 ▶); Jiang & Li (2004 ▶); Liu *et al.* (2004 ▶).
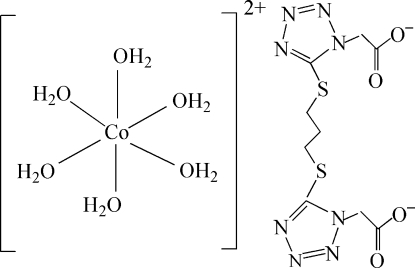


## Experimental

### 

#### Crystal data

[Co(H_2_O)_6_](C_9_H_10_N_8_O_4_S_2_)
*M* *_r_* = 525.42
Monoclinic, 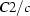

*a* = 19.420 (2) Å
*b* = 7.9069 (11) Å
*c* = 13.7356 (17) Å
β = 112.957 (2)°
*V* = 1942.1 (4) Å^3^
*Z* = 4
Mo *K*α radiation
μ = 1.17 mm^−1^
*T* = 298 K
0.38 × 0.35 × 0.20 mm

#### Data collection

Bruker SMART CCD area-detector diffractometer
Absorption correction: multi-scan (*SADABS*; Bruker, 1997 ▶) *T*_min_ = 0.665, *T*_max_ = 0.800
4714 measured reflections
1716 independent reflections
1518 reflections with *I* > 2σ(*I*)
*R*_int_ = 0.024

#### Refinement

*R*[*F* ^2^ > 2σ(*F* ^2^)] = 0.025
*wR*(*F* ^2^) = 0.068
*S* = 1.02
1716 reflections
142 parameters
H atoms treated by a mixture of independent and constrained refinement
Δρ_max_ = 0.24 e Å^−3^
Δρ_min_ = −0.36 e Å^−3^

### 

Data collection: *SMART* (Bruker, 1997 ▶); cell refinement: *SAINT* (Bruker, 1997 ▶); data reduction: *SAINT*; program(s) used to solve structure: *SHELXS97* (Sheldrick, 2008 ▶); program(s) used to refine structure: *SHELXL97* (Sheldrick, 2008 ▶); molecular graphics: *SHELXTL* (Sheldrick, 2008 ▶); software used to prepare material for publication: *SHELXTL* and *PLATON* (Spek, 2009 ▶).

## Supplementary Material

Crystal structure: contains datablocks I, global. DOI: 10.1107/S1600536809028463/hk2740sup1.cif

Structure factors: contains datablocks I. DOI: 10.1107/S1600536809028463/hk2740Isup2.hkl

Additional supplementary materials:  crystallographic information; 3D view; checkCIF report

## Figures and Tables

**Table 1:** Hydrogen-bond geometry (Å, °)

*D*—H⋯*A*	*D*—H	H⋯*A*	*D*⋯*A*	*D*—H⋯*A*
O3—H3*A*⋯O1	0.85	1.93	2.767 (2)	166
O4—H4*A*⋯O2^i^	0.85	1.90	2.742 (2)	172
O4—H4*B*⋯N3^ii^	0.85	2.19	2.970 (2)	152
O5—H5*A*⋯O1^i^	0.85	1.86	2.7069 (19)	173
O5—H5*B*⋯N4^iii^	0.85	1.99	2.833 (2)	173

Symmetry codes: (i) 
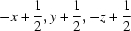
; (ii) 
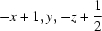
; (iii) 
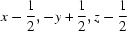
.

**Fig. 1.**
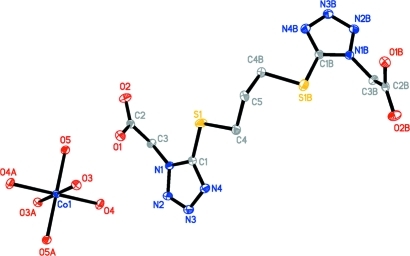
The molecular structure of the title molecule with the atom-numbering scheme. Displacement ellipsoids are drawn at the 30% probability level [symmetry codes: (A) 1/2 - x, 1/2 - y, -z; (B) 1 - x, y, 3/2 -z]. Hydrogen atoms have been omitted for clarity.

**Fig. 2.**
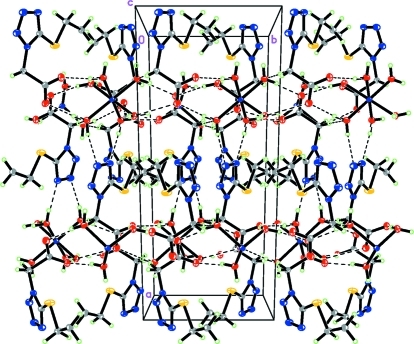
A partial packing diagram. Hydrogen bonds are shown as dashed lines.

## supplementary crystallographic information

### Comment

Coordination compounds of the type [Co(H_2_O)_6_](C_10_H_8_O_6_), [Co(H_2_O)_6_](*L*)_2_ (*L* = isonicotinate N-oxide, C_6_H_4_NO_3_) and [Co(H_2_O)_6_].2H_2_O.2*L*.2ClO_4_ (*L* = 1,1'-(propane -1,3-diyl)dipyridinium-4-carboxylate) have been previously prepared and studied by several groups (Liu *et al.*, 2004; Du *et al.*, 2004; Jiang & Li, 2004). We report herein the crystal structure of the new title mononuclear cobalt complex, [Co(H_2_O)_6_].(battp) [where battp is 1,3-bis(1-acetic acid-1,2,3,4-tetrazole-5- thioether propane)].

The asymmetric unit of the title complex contains one-half of the [Co(H_2_O)_6_]^2+^ cation and one-half of the [battp]^2-^ anion (Fig. 1). The Co center is coordinated by six H_2_O molecules in a distorted octahedral coordination environment. The average Co-O bond distance is 2.0973 (14) Å.

In the crystal structure, intra- and intermolecular O-H···O and O-H···N hydrogen bonds (Table 1) link the [Co(H_2_O)_6_]^2+^ cations and [battp]^2-^ anions into a three-dimensional network (Fig. 2), in which they may be effective in the stabilization of the structure. The π–π contact between the tetrazole rings, Cg1—Cg1^i^ [symmetry code: (i) -x, 2 - y, -z, where Cg1 is centroid of the ring A (N1-N4/C1)] may further stabilize the structure, with centroid-centroid distance of 3.346 (1) Å.

### Experimental

For the preparation of the title compound, H_2_battp (0.1440 g, 0.4 mmol) was dissolved in water (5 ml), and the solution of CoClO_4_.6H_2_O (0.1832 g, 0.5 mmol) in distilled water (5 ml) was added. The mixture was stirred at 353 K for 3 h, and then cooled and filtered. The filtrate was allowed to slowly evaporate at room temperature. Two weeks later, pink block crystals were obtained.

### Refinement

Atom H5 was located in difference Fourier map and refined isotropically. The remaining H atoms were positioned geometrically with O-H = 0.85 Å (for H_2_O) and C-H = 0.97 Å for methylene H atoms, respectively, and constrained to ride on their parent atoms, with U_iso_(H) = 1.2U_eq_(C,O).

### Crystal data

**Table:**

[Co(H_2_O)_6_](C_9_H_10_N_8_O_4_S_2_)	*F*(000) = 1084
*M**_r_* = 525.42	*D*_x_ = 1.797 Mg m^−^^3^
Monoclinic, *C*2/*c*	Mo *K*α radiation, λ = 0.71073 Å
Hall symbol: -C 2yc	Cell parameters from 3230 reflections
*a* = 19.420 (2) Å	θ = 2.8–28.0°
*b* = 7.9069 (11) Å	µ = 1.17 mm^−^^1^
*c* = 13.7356 (17) Å	*T* = 298 K
β = 112.957 (2)°	Block, pink
*V* = 1942.1 (4) Å^3^	0.38 × 0.35 × 0.20 mm
*Z* = 4	

### Data collection

**Table:**

Bruker SMART CCD area-detector diffractometer	1716 independent reflections
Radiation source: fine-focus sealed tube	1518 reflections with *I* > 2σ(*I*)
graphite	*R*_int_ = 0.024
φ and ω scans	θ_max_ = 25.0°, θ_min_ = 2.3°
Absorption correction: multi-scan (*SADABS*; Bruker, 1997)	*h* = −22→22
*T*_min_ = 0.665, *T*_max_ = 0.800	*k* = −5→9
4714 measured reflections	*l* = −16→16

### Refinement

**Table:**

Refinement on *F*^2^	Primary atom site location: structure-invariant direct methods
Least-squares matrix: full	Secondary atom site location: difference Fourier map
*R*[*F*^2^ > 2σ(*F*^2^)] = 0.025	Hydrogen site location: inferred from neighbouring sites
*wR*(*F*^2^) = 0.068	H atoms treated by a mixture of independent and constrained refinement
*S* = 1.02	*w* = 1/[σ^2^(*F*_o_^2^) + (0.0317*P*)^2^ + 3*P*] where *P* = (*F*_o_^2^ + 2*F*_c_^2^)/3
1716 reflections	(Δ/σ)_max_ < 0.001
142 parameters	Δρ_max_ = 0.24 e Å^−^^3^
0 restraints	Δρ_min_ = −0.36 e Å^−^^3^

### Special details

**Table:**

Geometry. All e.s.d.'s (except the e.s.d. in the dihedral angle between two l.s. planes) are estimated using the full covariance matrix. The cell e.s.d.'s are taken into account individually in the estimation of e.s.d.'s in distances, angles and torsion angles; correlations between e.s.d.'s in cell parameters are only used when they are defined by crystal symmetry. An approximate (isotropic) treatment of cell e.s.d.'s is used for estimating e.s.d.'s involving l.s. planes.
Refinement. Refinement of *F*^2^ against ALL reflections. The weighted *R*-factor *wR* and goodness of fit *S* are based on *F*^2^, conventional *R*-factors *R* are based on *F*, with *F* set to zero for negative *F*^2^. The threshold expression of *F*^2^ > σ(*F*^2^) is used only for calculating *R*-factors(gt) *etc*. and is not relevant to the choice of reflections for refinement. *R*-factors based on *F*^2^ are statistically about twice as large as those based on *F*, and *R*- factors based on ALL data will be even larger.

### Fractional atomic coordinates and isotropic or equivalent isotropic displacement parameters (Å^2^)

**Table:**

	*x*	*y*	*z*	*U*_iso_*/*U*_eq_	
Co1	0.2500	0.2500	0.0000	0.01988 (13)	
S1	0.43307 (3)	0.19008 (9)	0.57138 (5)	0.03941 (18)	
O1	0.31180 (8)	0.09876 (18)	0.31741 (11)	0.0285 (3)	
O2	0.23139 (9)	0.23926 (18)	0.36776 (14)	0.0344 (4)	
O3	0.30995 (8)	0.07215 (18)	0.11553 (11)	0.0263 (3)	
H3A	0.3132	0.0970	0.1774	0.032*	
H3B	0.3001	−0.0328	0.1066	0.032*	
O4	0.32343 (8)	0.43840 (19)	0.09350 (12)	0.0298 (3)	
H4A	0.3105	0.5351	0.1074	0.036*	
H4B	0.3708	0.4302	0.1214	0.036*	
O5	0.18294 (8)	0.30070 (18)	0.08298 (11)	0.0262 (3)	
H5A	0.1885	0.3941	0.1158	0.031*	
H5B	0.1362	0.2841	0.0519	0.031*	
N1	0.41422 (9)	0.3568 (2)	0.39209 (13)	0.0222 (4)	
N2	0.45252 (10)	0.4106 (2)	0.33400 (14)	0.0284 (4)	
N3	0.52125 (10)	0.3673 (2)	0.38552 (14)	0.0314 (4)	
N4	0.52993 (10)	0.2844 (2)	0.47718 (14)	0.0287 (4)	
C1	0.46251 (12)	0.2798 (3)	0.47931 (16)	0.0237 (4)	
C2	0.28859 (12)	0.2281 (2)	0.34801 (15)	0.0225 (4)	
C3	0.33493 (11)	0.3904 (3)	0.36361 (17)	0.0246 (4)	
H3C	0.3160	0.4558	0.2988	0.030*	
H3D	0.3290	0.4578	0.4188	0.030*	
C4	0.52031 (11)	0.1138 (3)	0.67099 (16)	0.0282 (5)	
H4C	0.5467	0.0439	0.6387	0.034*	
H4D	0.5522	0.2081	0.7063	0.034*	
C5	0.5000	0.0101 (4)	0.7500	0.0268 (6)	
H5	0.5438 (12)	−0.061 (3)	0.7883 (18)	0.033 (6)*	

### Atomic displacement parameters (Å^2^)

**Table:**

	*U*^11^	*U*^22^	*U*^33^	*U*^12^	*U*^13^	*U*^23^
Co1	0.0184 (2)	0.0201 (2)	0.0220 (2)	0.00018 (14)	0.00878 (16)	−0.00122 (15)
S1	0.0235 (3)	0.0601 (4)	0.0348 (3)	0.0030 (3)	0.0115 (3)	0.0230 (3)
O1	0.0354 (9)	0.0223 (8)	0.0279 (8)	0.0004 (6)	0.0124 (7)	0.0000 (6)
O2	0.0315 (9)	0.0284 (9)	0.0507 (10)	−0.0033 (7)	0.0239 (8)	0.0010 (7)
O3	0.0293 (8)	0.0234 (7)	0.0267 (8)	0.0031 (6)	0.0115 (6)	0.0008 (6)
O4	0.0196 (7)	0.0275 (8)	0.0386 (9)	−0.0016 (6)	0.0073 (6)	−0.0095 (7)
O5	0.0225 (7)	0.0268 (8)	0.0313 (8)	−0.0010 (6)	0.0126 (6)	−0.0066 (6)
N1	0.0205 (9)	0.0233 (9)	0.0236 (9)	−0.0013 (7)	0.0096 (7)	0.0014 (7)
N2	0.0279 (10)	0.0347 (10)	0.0260 (9)	−0.0019 (8)	0.0141 (8)	0.0019 (8)
N3	0.0265 (10)	0.0405 (11)	0.0302 (10)	−0.0008 (8)	0.0144 (8)	0.0018 (8)
N4	0.0228 (9)	0.0340 (10)	0.0300 (10)	−0.0008 (8)	0.0112 (8)	0.0010 (8)
C1	0.0231 (11)	0.0255 (11)	0.0225 (10)	−0.0012 (8)	0.0089 (9)	−0.0006 (8)
C2	0.0243 (11)	0.0232 (11)	0.0179 (10)	0.0008 (8)	0.0059 (8)	0.0040 (8)
C3	0.0204 (10)	0.0223 (10)	0.0304 (11)	0.0019 (8)	0.0090 (9)	0.0018 (9)
C4	0.0231 (11)	0.0357 (12)	0.0236 (10)	0.0023 (9)	0.0066 (9)	0.0007 (9)
C5	0.0282 (17)	0.0277 (16)	0.0211 (15)	0.000	0.0061 (13)	0.000

### Geometric parameters (Å, °)

**Table:**

Co1—O3	2.1000 (14)	O5—H5B	0.8500
Co1—O3^i^	2.1000 (14)	N1—N2	1.354 (2)
Co1—O4^i^	2.1150 (14)	N1—C1	1.345 (3)
Co1—O4	2.1150 (14)	N1—C3	1.458 (3)
Co1—O5^i^	2.0770 (13)	N2—N3	1.290 (2)
Co1—O5	2.0770 (13)	N3—N4	1.370 (3)
S1—C1	1.730 (2)	N4—C1	1.321 (3)
S1—C4	1.816 (2)	C2—C3	1.533 (3)
O1—C2	1.254 (2)	C3—H3C	0.9700
O2—C2	1.245 (3)	C3—H3D	0.9700
O3—H3A	0.8500	C4—C5	1.529 (3)
O3—H3B	0.8500	C4—H4C	0.9700
O4—H4A	0.8499	C4—H4D	0.9700
O4—H4B	0.8499	C5—C4^ii^	1.529 (3)
O5—H5A	0.8500	C5—H5	0.98 (2)
			
O3—Co1—O3^i^	180.00 (12)	H4A—O4—H4B	109.2
O3—Co1—O4^i^	91.78 (6)	Co1—O5—H5A	118.7
O3^i^—Co1—O4^i^	88.22 (6)	Co1—O5—H5B	118.1
O3—Co1—O4	88.22 (6)	H5A—O5—H5B	106.9
O3^i^—Co1—O4	91.78 (6)	C1—S1—C4	102.11 (10)
O4^i^—Co1—O4	180.00 (14)	N4—C1—N1	108.69 (18)
O5^i^—Co1—O3	90.23 (6)	N4—C1—S1	129.81 (17)
O5—Co1—O3	89.77 (6)	N1—C1—S1	121.49 (16)
O5^i^—Co1—O3^i^	89.77 (6)	O2—C2—O1	126.76 (19)
O5—Co1—O3^i^	90.23 (6)	O2—C2—C3	115.80 (18)
O5^i^—Co1—O4^i^	87.10 (6)	O1—C2—C3	117.44 (18)
O5—Co1—O4^i^	92.90 (6)	N1—C3—C2	112.70 (16)
O5^i^—Co1—O4	92.90 (6)	N1—C3—H3C	109.1
O5—Co1—O4	87.10 (6)	C2—C3—H3C	109.1
O5^i^—Co1—O5	180.00 (10)	N1—C3—H3D	109.1
C1—N1—N2	108.35 (16)	C2—C3—H3D	109.1
C1—N1—C3	128.18 (17)	H3C—C3—H3D	107.8
N2—N1—C3	123.36 (16)	S1—C4—H4C	110.4
N3—N2—N1	106.36 (16)	S1—C4—H4D	110.4
N2—N3—N4	111.19 (16)	C5—C4—S1	106.84 (12)
C1—N4—N3	105.41 (17)	C5—C4—H4C	110.4
Co1—O3—H3A	113.8	C5—C4—H4D	110.4
Co1—O3—H3B	121.1	H4C—C4—H4D	108.6
H3A—O3—H3B	107.2	C4—C5—C4^ii^	115.2 (3)
Co1—O4—H4A	125.6	C4—C5—H5	106.0 (13)
Co1—O4—H4B	125.1	C4^ii^—C5—H5	109.5 (13)
			
C1—N1—N2—N3	−0.2 (2)	C3—N1—C1—S1	5.2 (3)
C3—N1—N2—N3	176.28 (18)	C4—S1—C1—N4	3.5 (2)
N1—N2—N3—N4	0.3 (2)	C4—S1—C1—N1	−178.16 (17)
N2—N3—N4—C1	−0.2 (2)	C1—N1—C3—C2	−63.3 (3)
N3—N4—C1—N1	0.1 (2)	N2—N1—C3—C2	120.9 (2)
N3—N4—C1—S1	178.55 (17)	O2—C2—C3—N1	150.83 (18)
N2—N1—C1—N4	0.1 (2)	O1—C2—C3—N1	−28.9 (3)
C3—N1—C1—N4	−176.20 (19)	C1—S1—C4—C5	−172.50 (15)
N2—N1—C1—S1	−178.54 (15)	S1—C4—C5—C4^ii^	−77.35 (11)

Symmetry codes: (i) −*x*+1/2, −*y*+1/2, −*z*; (ii) −*x*+1, *y*, −*z*+3/2.

### Hydrogen-bond geometry (Å, °)

**Table:**

*D*—H···*A*	*D*—H	H···*A*	*D*···*A*	*D*—H···*A*
O3—H3A···O1	0.85	1.93	2.767 (2)	166
O4—H4A···O2^iii^	0.85	1.90	2.742 (2)	172
O4—H4B···N3^iv^	0.85	2.19	2.970 (2)	152
O5—H5A···O1^iii^	0.85	1.86	2.7069 (19)	173
O5—H5B···N4^v^	0.85	1.99	2.833 (2)	173

Symmetry codes: (iii) −*x*+1/2, *y*+1/2, −*z*+1/2; (iv) −*x*+1, *y*, −*z*+1/2; (v) *x*−1/2, −*y*+1/2, *z*−1/2.
